# Ringo – an R/Bioconductor package for analyzing ChIP-chip readouts

**DOI:** 10.1186/1471-2105-8-221

**Published:** 2007-06-26

**Authors:** Joern Toedling, Oleg Sklyar, Wolfgang Huber

**Affiliations:** 1 EMBL European Bioinformatics Institute, Wellcome Trust Genome Campus, Hinxton, Cambridge CB10 1SD, UK

## Abstract

**Background:**

Chromatin immunoprecipitation combined with DNA microarrays (*ChIP-chip*) is a high-throughput assay for DNA-protein-binding or post-translational chromatin/histone modifications. However, the raw microarray intensity readings themselves are not immediately useful to researchers, but require a number of bioinformatic analysis steps. Identified enriched regions need to be bioinformatically annotated and compared to related datasets by statistical methods.

**Results:**

We present a free, open-source R package *Ringo *that facilitates the analysis of ChIP-chip experiments by providing functionality for data import, quality assessment, normalization and visualization of the data, and the detection of ChIP-enriched genomic regions.

**Conclusion:**

*Ringo *integrates with other packages of the Bioconductor project, uses common data structures and is accompanied by ample documentation. It facilitates the construction of programmed analysis workflows, offers benefits in scalability, reproducibility and methodical scope of the analyses and opens up a broad selection of follow-up statistical and bioinformatic methods.

## Background

Chromatin immunoprecipitation followed by DNA microarray hybridization (*ChIP-chip*) is a powerful technology for the systematic identification of genomic sites at which transcription factors bind or histone proteins bear post-translational modifications [1]. The raw microarray intensity readings themselves are not immediately useful to researchers, though. Through a number of bioinformatic analysis steps, one can obtain from the raw data a processed list of genomic sites and quantitative measures such as strength of evidence for a site, its extent, and estimates of relative occupancy.

We provide a freely available, open-source software module *Ringo *for the import of the raw microarray data, their quality assessment, normalization, visualization, and for the detection and quantitation of ChIP-enriched regions. Its functionality covers the complete primary analysis for ChIP-chip tiling microarrays, especially those from the company NimbleGen. *Ringo *is integrated with the Bioconductor [2] project of bioinformatic extension packages to the R statistical software. This design makes it easy for users to construct sophisticated analyses approaches that also leverage other R/Bioconductor functionality, for example additional normalization methods from the *affy *[3] and *oligo *packages, or wavelet analysis methods from R's signal processing packages.

*Ringo *is complementary to existing available software for ChIP microarray analysis. For example, mpeak [4], TiMAT , MAT [5], TileMap [6], ACME [7], HGMM [8], and ChIPOTle [9] provide powerful model-based and non-parametric algorithms for finding ChIP-enriched regions on normalized and quality controlled ChIP-chip data. A focus of these softwares has been to provide easy-to-use interfaces to these algorithms, and users are asked to use them in combination with other tools for the data import, preprocessing and follow-up statistical and bioinformatic analysis. A unique aspect of *Ringo *is that it facilitates the construction of more automated programmed workflows and offers benefits in the scalability, reproducibility and methodical scope of the analyses.

## Implementation

*Ringo *is an extension package for the programming language and statistical environment R [10]. Most of its functionality is also implemented in R, for some performance-critical computations C++ functions are used. The package has been developed to analyze two-color ChIP-chip oligonucleotide microarrays from the company NimbleGen (for NimbleGen one-color microarrays, we recommend the Bioconductor package *oligo*). Analogous two-color tiling array platforms from other vendors can also be processed. The package employs functions from other packages of the Bioconductor project [2], most notably from the package *limma *[11], It employs object classes that are also standard in other Bioconductor packages, such as *limma*'s RGList and *Biobase*'s ExpressionSet, and provides a new object class for representing identified ChIP-enriched regions.

## Results and discussion

**Figure 1:**
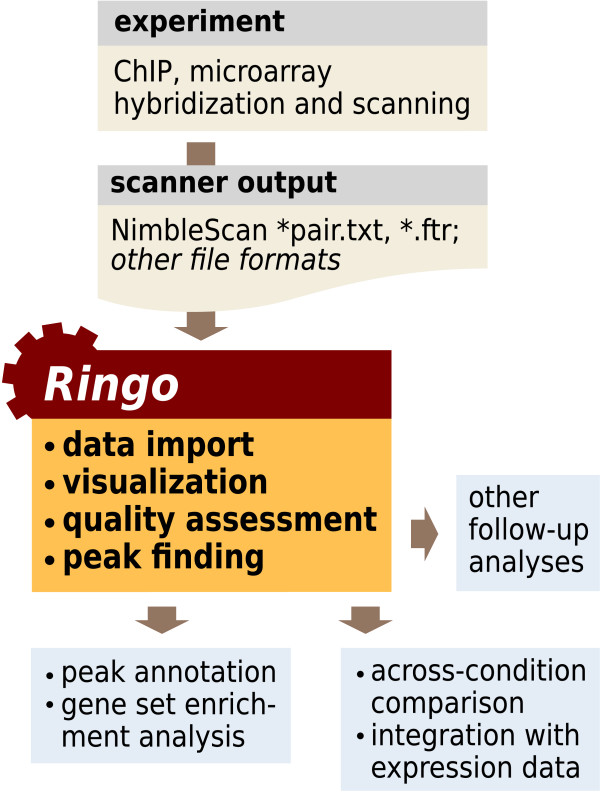
**ChIP-chip analysis with Ringo**. Workflow diagram displaying which steps of the analysis of ChIP-chip experiments are facilitated by *Ringo*.

Figure 1 shows a typical workflow of the analysis of ChIP-chip experiments and indicates which steps are facilitated by the Bioconductor package *Ringo*. Key functionalities of *Ringo *are import, quality assessment and preprocessing of the raw data, visualization of the raw and processed data and a detection algorithm for enrichment peaks.

The package contains functions to read in the raw NimbleScan output files of the microarrays into an RGList object. The user can alternatively choose to supply other raw microarray data in RGList format. Such an object is essentially a list and contains the raw intensities of the two hybridizations for the red and green channel plus information on the probes on the array and on the analyzed samples.

*Ringo *contains an extensive set of functions for quality assessment of the data (see, e.g., [12] for an overview of quality assessment methods in the context of two-color microarray data).

Its image function allows one to look at the spatial distribution of the intensities on a chip. This can be useful to detect obvious artifacts on the array, such as scratches, bright spots, finger prints etc. that might render parts or all of the readouts invalid.

**Figure 2:**
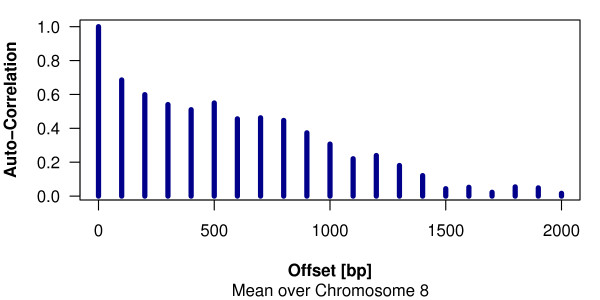
**Autocorrelation in ChIP-chip data**. An example data set on histone-3-acetylation, which is provided as part of the software documentation, is used to demonstrate the package's autocorrelation plot. For each base-pair offset *d*, it assesses how strong the intensities of probes mapped to genomic positions *x *+ *d *are correlated with the probe intensities at positions *x*. The computed correlation is plotted against the offset.

To assess whether probe tiling across the chromosome affects levels of close-by probes, one can look at the *autocorrelation *plot. For each base-pair offset *d*, it is assessed how strong the intensities of probes at genomic positions *x *+ *d *are correlated with the probe intensities at positions *x*. The computed correlation is plotted against the offset *d *(see Figure 2). For regions that are present in the immuno-precipitate or in the genomic *input *sample, high autocorrelation is to be expected in a range corresponding to the size distribution of the fragmented DNA. The autocorrelation is relevant for the quality assessment of data and has to be taken into account in subsequent statistical analyses.

Furthermore, if the data set contains biological or technical replicates, low correlation between replicate samples' intensities may indicate microarrays of questionable quality. *Ringo *therefore contains functions to visualize the correlation between replicate samples' raw and preprocessed intensities.

Following quality assessment of the data, one usually aims to increase the signal-to-noise ratio of the data by *normalization *of the probe intensities and derive fold changes of probes' intensities in the enriched sample divided by their intensities in the non-enriched input sample and take the (generalized) logarithm of these ratios.

For normalization, *Ringo *provides a number of choices, interfacing preprocessing methods implemented in the Bioconductor packages *vsn *[13] and *limma *plus the Tukey-biweight scaling of the log-ratios that is suggested by NimbleGen. The normalization procedure results in an ExpressionSet object of normalized probe levels, the basic Bioconductor object class for microarray data, with which many other Bioconductor packages can easily interact.

In addition, a mapping between probes on the microarray and genomic positions is required. *Ringo *uses a set of tables relating chromosomal positions to feature identifiers on the array. The package provides scripts that assist in the production of such a table from either a NimbleGen POS file or, what is often preferable, custom alignments of the probe sequences to the genome of interest.

**Figure 3:**
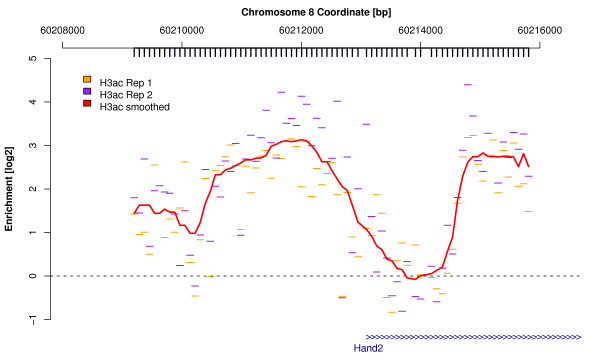
**Visualization of ChIP-enriched genomic regions**. Original and smoothed probe-wise fold-changes for histone-3-acetylation (H3ac) in the vicinity of the transcription start site of the *Hand2 *gene on chromosome 8. The bold ticks beneath the genomic coordinate axis indicate genomic positions at which microarray probes target the genome sequence.

An important aspect of genomic data analysis is a thorough observation of as many data examples as possible using a number of different visualization techniques. In addition to the multitude of visualization functions offered by other R and Bioconductor packages, *Ringo *provides a function to display estimates of log fold enrichment with probes mapped to matching chromosomal positions (see Figure 3).

On the normalized probe levels, one next aims to identify those genomic regions that show enrichment in the immuno-precipitated sample as compared to the untreated input sample. *Ringo *contains a heuristic algorithm that we developed to identify genomic regions bearing certain histone modifications. The details of this algorithm are described in the package vignette. It is built upon a *smoothing *procedure: smoothing across genomically neighboring probes is often employed to ameliorate probe-specific variability in the data, that is, the effect that different probes measure the same target DNA amount with different efficiency. This may be caused by different qualities of probe synthesis on the array, probe GC content, target cDNA secondary structure, cross-hybridization, and other reasons. An important issue with enrichment detection procedures is *background *signal: some non-antibody-bound DNA may be pulled down during the immuno-precipitation and consequently enriched, potentially resulting in false positives. The enrichment detection algorithm in *Ringo *is a first and sufficiently effective approach to these problems, but more research is necessary to establish optimal methodology.

## Conclusion

The functionality of the software package *Ringo *provides a good starting point for researchers interested in the analysis of NimbleGen ChIP microarrays or of similar data. It is an add-on package for the widely used programming language and statistical environment R, and integrates with the Bioconductor project of bioinformatic R extension packages.

As other Bioconductor/R packages, *Ringo *offers a high level of documentation through its vignette and the function help pages, and the access to the documentation is standardized. Furthermore, also the distribution, installation and maintenance of the packages are standardized, and responsive and competent user support is provided through the Bioconductor mailing list. These features are often difficult to find with free, open-source software.

*Ringo *provides a comprehensive set of functions for quality assessment, data processing, visualization and ChIP-chip data analysis. The package's close integration with other Bioconductor packages opens up a multitude of subsequent analysis approaches.

## Availability and requirements

The R-package *Ringo *is available from the Bioconductor web site at  and runs on Linux, Mac OS and MS-Windows. It requires an installed version of R (version ≥ 2.5.0), which is freely available from the Comprehensive R Archive Network (CRAN) at , and other Bioconductor packages, namely *Biobase*, *affy*, *geneplotter*, *limma*, and *vsn *plus the CRAN package *RColorBrewer*. The easiest way to obtain the most recent version of the software, with all its dependencies, is to follow the instructions at . *Ringo *is distributed under the terms of the Artistic License 2.0.

## Authors' contributions

All authors contributed significantly to the final version of the software package. JT wrote the manuscript. All authors read and approved of the final version of the manuscript.
