# Ringo – an R/Bioconductor package for analyzing ChIP-chip readouts

**DOI:** 10.1186/1471-2105-8-443

**Published:** 2007-11-15

**Authors:** Joern Toedling, Oleg Skylar, Tammo Krueger, Jenny J Fischer, Silke Sperling, Wolfgang Huber

**Affiliations:** 1 EMBL European Bioinformatics Institute, Wellcome Trust Genome Campus, Hixton, Cambridge CB10 1SD, UK; 2 Max Planck Institute for Molecular Genetics, Berlin, Germany

## Correction

Following the publication of the article "Ringo – an R/Bioconductor package for analyzing ChIP-chip readouts – *BMC Bioinformatics *2007, **8:**221" [1], the submitting author became aware that co-authors had been omitted from the publication.

Therefore, this article has been submitted as a correction to the original text, and highlights the contributions that these authors made to the original article.

The author would like to apologise for any inconvenience this may have caused.

## Corrected author list

Joern Toedling, Oleg Skylar, Tammo Krueger, Jenny J. Fischer, Silke Sperling and Wolfgang Huber.

## Corrected authors' contributions

JT, OS and TK developed the software. JT wrote the manuscript. JJF and SS provided example data. All authors contributed to design of analytical algorithms. All authors read and approved the final manuscript.
